# LncRNA HMMR-AS1 promotes proliferation and metastasis of lung adenocarcinoma by regulating MiR-138/sirt6 axis

**DOI:** 10.18632/aging.101958

**Published:** 2019-05-25

**Authors:** Yong Cai, Zhaoying Sheng, Yun Chen, Jiying Wang

**Affiliations:** 1 Department of Radiation Oncology, Shanghai Pulmonary Hospital, Tongji University School of Medicine, Shanghai 200433, China; 2 Department of Oncology, Shanghai Pulmonary Hospital, Tongji University School of Medicine, Shanghai 200433, China; * Equal Contribution

**Keywords:** lung adenocarcinoma (LUAD), HMMR-AS1, miR-138, sirt6, proliferation, apoptosis, tumorigenesis

## Abstract

Purpose: Long noncoding RNAs (lncRNA) play critical roles in cancer development. In this study, we aimed to explore the function and possible molecular mechanism of HMMR-AS1 involved in lung adenocarcinoma (LUAD).

Experimental Design: Firstly, we analyzed HMMR-AS1 expression in LUAD tissues with the sequencing data from The Cancer Genome Atlas (TCGA). Next, we evaluated the effects of HMMR-AS1 on LUAD cell proliferation and apoptosis, and its regulation of miR-138 by acting as a ceRNA. The animal model was used to support the *in vitro* experimental findings.

Results: HMMR-AS1 expression was significantly upregulated in LUAD tissues and was associated with larger tumor diameter, advanced TNM stage, lymph node metastasis, and shorter survival. Knockdown of HMMR-AS1 induced apoptosis and growth arrest *in vitro* and inhibited tumorigenesis in mouse xenografts. Mechanistically, HMMR-AS1 functioned as a ceRNA of miR-138, thereby leading to repression of its endogenous target sirt6. Moreover, knockdown of HMMR-AS1 dramatically inhibited tumor growth and metastasis of LUAD *in vivo*.

Conclusions: Taken together, HMMR-AS1 is significantly over-expressed in LUAD, and HMMR-AS1–miR-138–sirt6 axis play a critical role in LUAD tumorigenesis. Our findings highlight an oncogenic role of HMMR-AS1 in LUAD.

## Introduction

Lung adenocarcinoma (LUAD) is the most common histological subtype of non-small-cell lung cancer [1,2]. Up to date, LUAD has become one of the most common histological subtypes instead of squamous cell carcinoma in many countries [3]. Although there are various ways for treatment of LUAD, the mortality rate has not been significantly improved yet [4]. Therefore, it’s critical to find novel therapeutic targets for improving the treatment.

Long non-coding RNAs (LncRNAs), are non-protein-encoding RNAs which are longer than 200 nucleotides, play important roles in the regulation of pathological and physiological processes in various types of cancer [5]. It has been reported that lncRNAs are involved in various significant cellular biological processes, including X-chromosome blots, stem cell differentiation, immune responses, cancer cell proliferation, as well as chemoresistance [6]. Recent reports have indicated that many lncRNAs, including PVT1, HOTAIR, LINC00673, ANRIL, HIT, and GAS5-AS1, are associated with tumors, particularly with lung cancer [7]. Currently, the clinical relevance, biological function, and potential mechanisms of several significant lncRNAs have been reported regarding LUAD [8,9]. SNHG3 plays a key role in LUAD via regulating RNA splicing, tRNA processing, signal transduction, cell adhesion, transcription, as well as apoptosis [10]. In addition, LINC00152 is highly expressed in LUAD tissues and cells, which promotes cancer cell proliferation and G1/S transition via interaction with EZH2 [11]. There is evidence that lncRNA plays an important role in LUAD, whereas the specific role of it in LUAD carcinogenesis is still unclear. Therefore, it is necessary to investigate the function of lncRNA and evaluate its potential as a diagnostic biomarker or therapeutic target for LUAD. Here, the function and molecular mechanism of HMMR-AS1 in LUAD progression. Our data suggest that HMMR-AS1 is a carcinogenic regulator in the pathogenesis of LUAD, which might be a candidate target in terms of LUAD diagnosis and treatment.

## RESULTS

### HMMR-AS1 is overexpressed in LUAD

**Figure 1:**
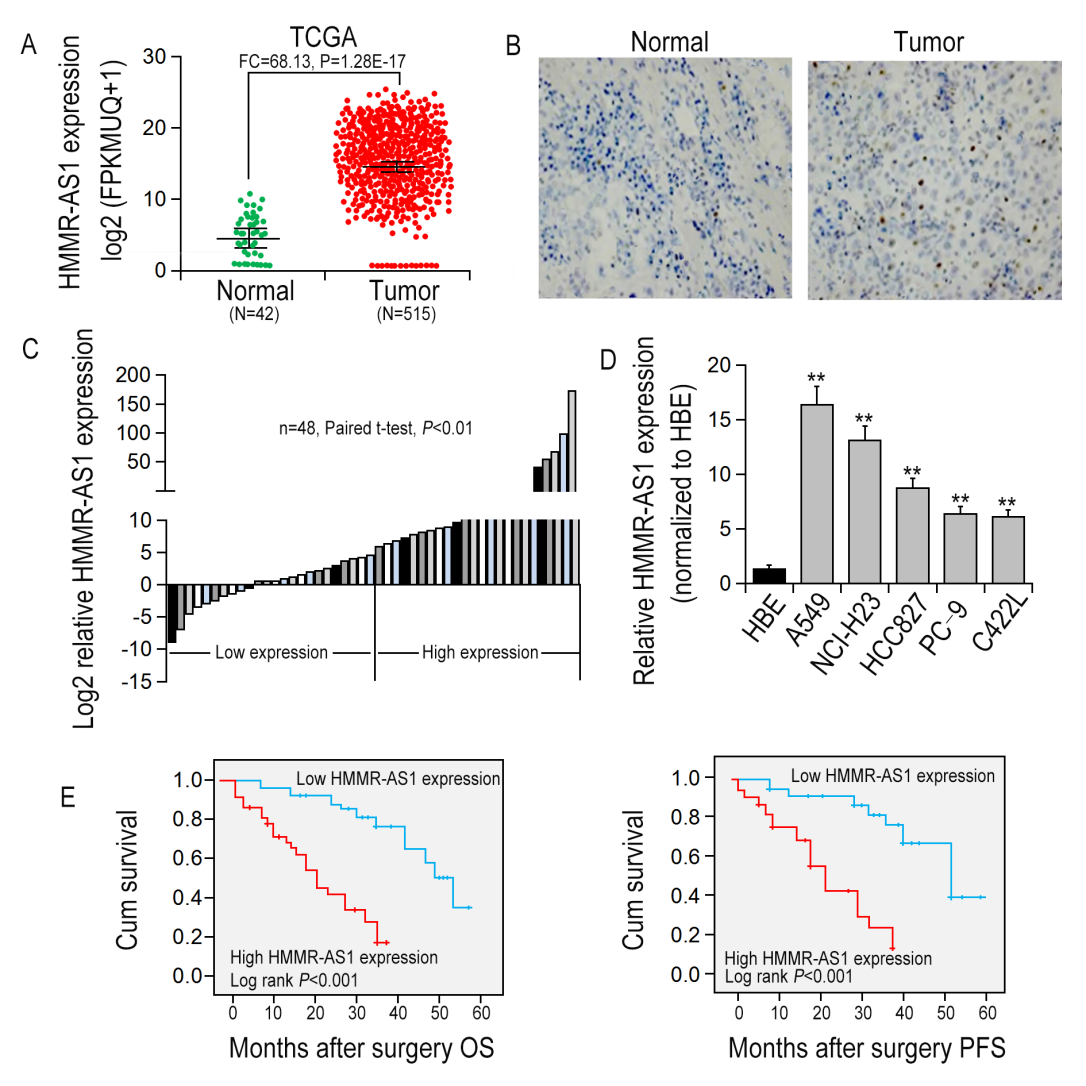
**HMMR-AS1 overexpression is associated with poor prognosis.** (**A**) Relative expression of HMMR-AS1 was analyzed by TCGA data. (**B**) HMMR-AS1 expressions in LUAD tissues and the adjacent para-carcinoma tissues were detected by *in situ* hybridization. (**C**) HMMR-AS1 expression in LUAD tissues was analyzed by qRT-PCR (n=48). (**D**) HMMR-AS1 expression in LUAD and HBE cells analyzed by qRT-PCR analysis. (**E, F**). Kaplan-Meier OS and DFS curves of patients with different HMMR-AS1 expression levels were plotted by the Kaplan-Meier method. *P < 0.05, **P < 0.01.

To uncover lncRNAs that might be involved in LUAD tumorgenesis, RNA sequencing data of 515 LUAD tissues (tumor group) from The Cancer Genome Atlas (TCGA) and 42 adjacent non-cancerous tissues (normal group) were analyzed. The result showed that HMMR-AS1 expression in tumor tissues was significantly higher than that in non-cancerous tissues (Fig. 1A). ISH assays with HMMR-AS1 probe also showed that HMMR-AS1 expression level was up-regulated in LUAD tissues, whereas it was relatively low in control tissues (Fig. 1B). In addition, we also detected HMMR-AS1 expression levels in 48 pairs of LUAD tissues as well as their matched non-cancerous tissues, and LUAD cell lines (A549, NCI-H23, HCC827, PC-9, and C422L) and HBE via qRT-PCR. The result showed that relative to adjacent non-cancerous tissues and HBE cell line, the expression of HMMR-AS1 in LUAD tissues and cells was significantly increased (Figures 1C-D). We analyzed the association of HMMR-AS1 expression levels with clinicopathological features of patients to assess the significance of HMMR-AS1 overexpression in LUAD in clinical practice. Median expression level was taken as a cut-off value and the 48 patients were divided into two groups, the low HMMR-AS1 expression group and the high HMMR-AS1 expression group. High expression of HMMR-AS1 was significantly associated with greater tumor volume (P = 0.038), depth of invasion (P = 0.026), as well as lymph node metastasis (P = 0.007, Supplementary Table S1). We also examined the involvement of HMMR-AS1 expression level in prognosis in patients with LUAD. Kaplan-Meier survival analysis uncovered that compared to patients with low HMMR-AS1 expression, OS and PFS were shorter in patients with high HMMR-AS1 expression (Fig. 1E).

### HMMR-AS1 silencing inhibits LUAD cell proliferation

**Figure 2:**
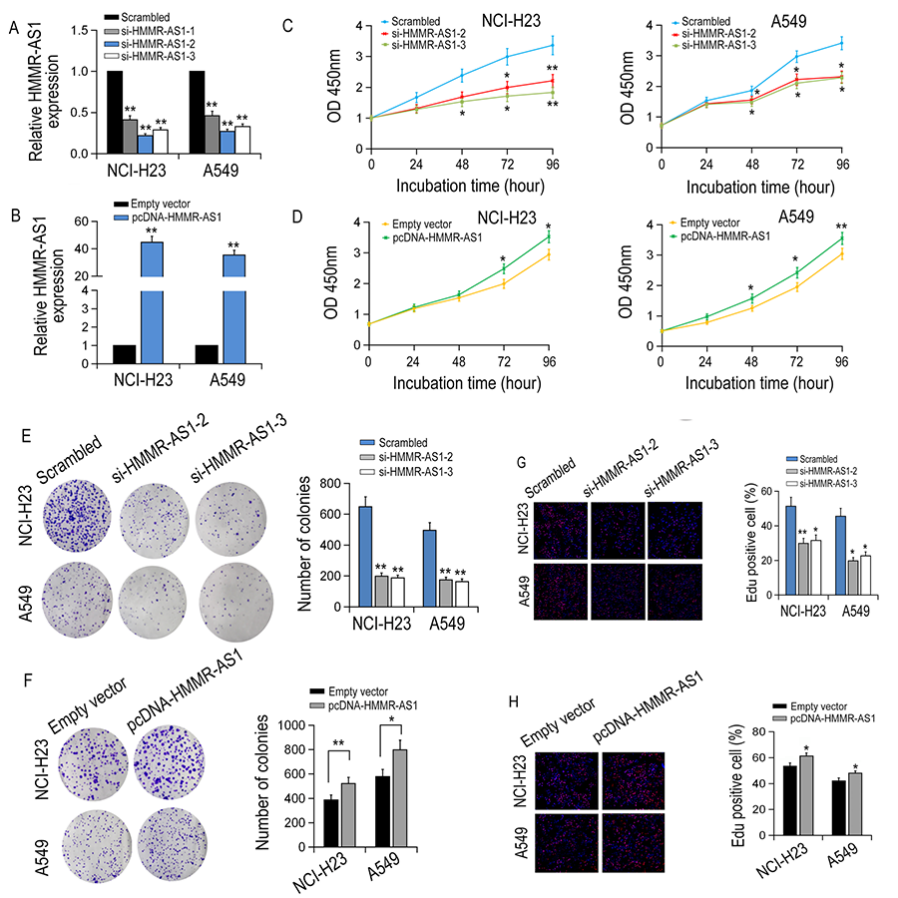
**The effect of HMMR-AS1 on LUAD cell proliferation *in vitro*.** (**A, B**) qRT-PCR analysis of HMMR-AS1 expression in transfected LUAD cells. (**C, D**) The viability of LUAD cells transfected with si-HMMR-AS1 or pcDNA-HMMR-AS1 were detected by CCK8 assays. (**E-H**) The proliferative rates of transfected LUAD cells determined by colony formation and EdU staining assay. *P < 0.05, **P < 0.01.

HMMR-AS1 expression was knocked down or overexpressed in NCI-H23 and A549 cells to investigate the biological function of HMMR-AS1 in LUAD cells (Fig. 2A-B). It was indicated that the cell proliferation of NCI-H23 and A549 cells was significantly inhibited by HMMR-AS1 knockdown, whereas the growth ability of NCI-H23 and A549 cells was promoted by HMMR-AS1 overexpression (Fig. 2C-D). Similarly, colony formation experiments also showed that the clone numbers of NCI-H23 and A549 cells were significantly reduced with HMMR-AS1 down-regulation but markedly increased with HMMR-AS1 up-regulation (Fig. 2E-F). Similar results were obtained in EdU proliferation assay (Fig. 2G-H).

### HMMR-AS1 knockdown induces apoptosis and cell cycle arrest in LUAD cells

**Figure 3:**
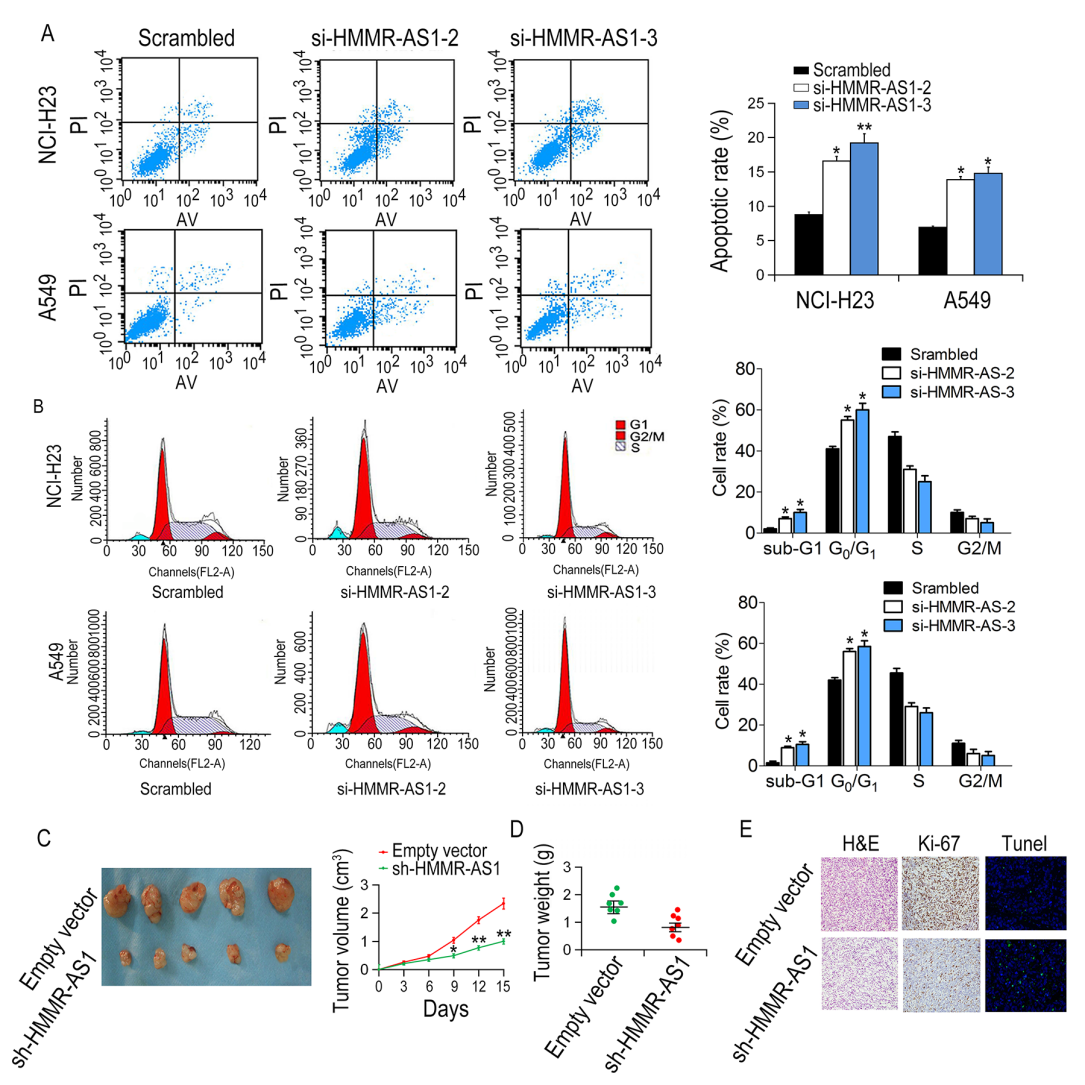
**Effects of HMMR-AS1 on proliferation, apoptosis, and cell cycle of LUAD cells *in vitro* and *in vivo*.** (**A, B**) The apoptotic rates and cell cycle of cells were determined by flow cytometry after HMMR-AS1 knockdown in NCI-H23 and A549 cells. (**C, D**). Tumor volume and weight were detected in tumor tissues of nude mice injected with HMMR-AS1 knockdown NCI-H23 cells. (**E**) Ki67 level and apoptosis were detected by TUNEL staining and immunohistochemistry, respectively. *P < 0.05, **P < 0.01.

Compared with the scrambled group, the apoptotic rate of NCI-H23 and A549 cells, transfected with si-HMMR-AS1-2 or si-HMMR-AS1-3 was increased (Fig. 3A). Moreover, cell cycle arrest was observed in NCI-H23 and A549 cells after transfection with si-HMMR-AS1 (Fig. 3B). These results indicate that inhibition of LUAD cell proliferation by HMMR-AS1 silencing is due to enhanced apoptosis and cell cycle arrest. The control shRNA or sh-HMMR-AS1, were stably transfected into NCI-H23 cells, which were injected subcutaneously into male nude mice to determine whether HMMR-AS1 was involved in tumor growth *in vivo*. Compared with the control group, the tumor size and volume were significantly reduced in the sh-HMMR-AS1 group (Fig. 3C-D). Immunohistochemical staining indicated that the expression of Ki-67 in sh-HMMR-AS1 group was lower compared to control group. TUNEL staining analysis indicated that the apoptotic rate of the sh-HMMR-AS1 group was significantly higher than that of the control group (Fig. 3E).

### HMMR-AS1 is a ceRNA and molecular sponge of miR-138 in LUAD cells

**Figure 4:**
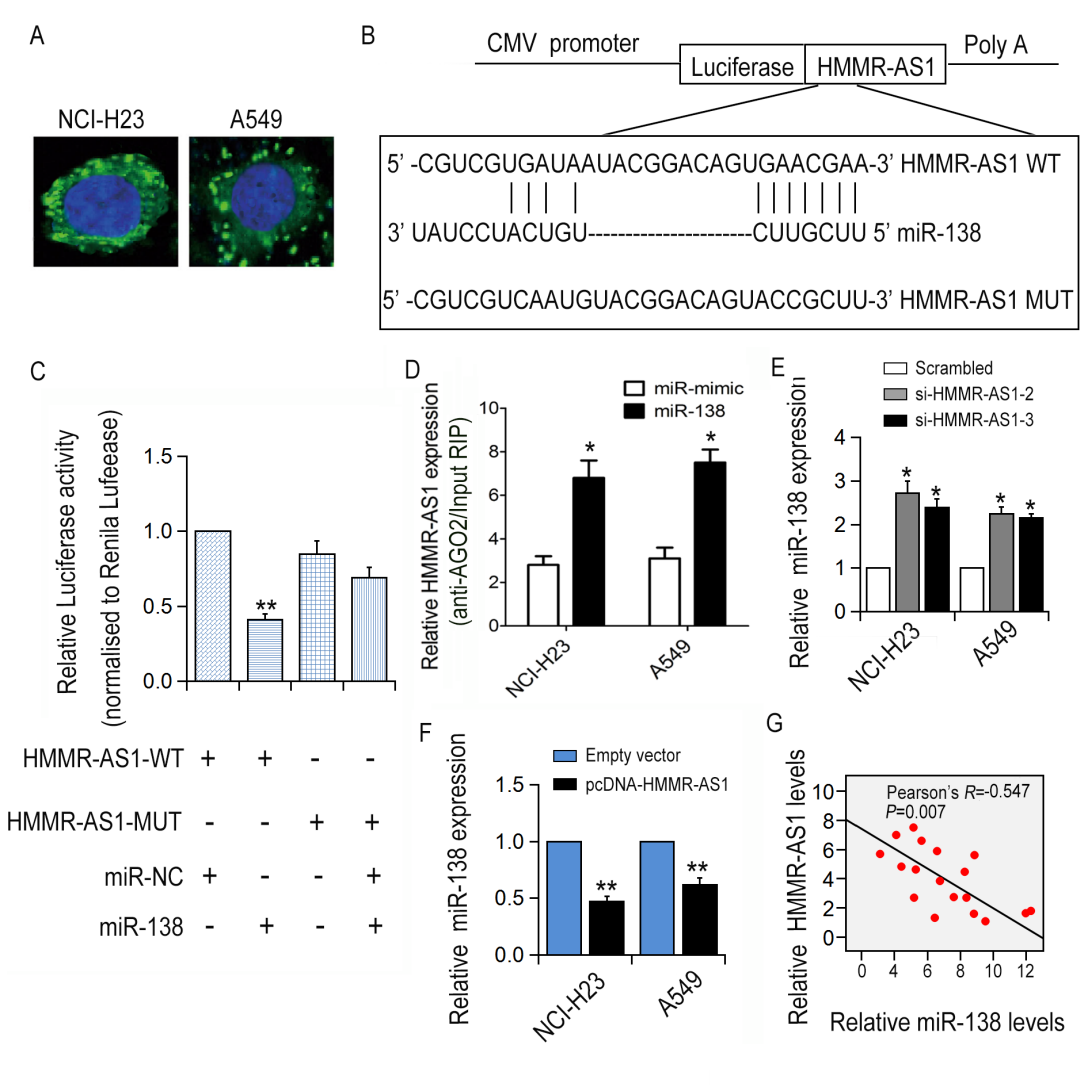
**The correlation between HMMR-AS1 and miR-138.** (**A**) HMMR-AS1 expression was detected in the cytoplasm (green) and nuclear fractions (blue) of NCI-H23 and A549 cells by FISH analysis. (**B, C**) The luciferase reporter plasmid containing WT/Mut HMMR-AS1 was co-transfected into HEK-293T cells. (**D**) RNA levels in immunoprecipitates are presented as fold enrichment in Ago2 relative to IgG immunoprecipitates. (**E, F**) miR-138 expression in NCI-H23 and A549 cells was detected by qRT-PCR analysis. (**G**) Correlation between HMMR-AS1 and miR-138 expression in 18 paired LUAD tissues. *P < 0.05, **P < 0.01.

FISH and subcellular fractionation assay results showed that HMMR-AS1 was abundantly expressed in the cytoplasm (Fig. 4A), indicating that HMMR-AS1 might regulate downstream gene expression at the post-transcriptional level. The luciferase reporter plasmid carrying the HMMR-AS1 sequence was transfected into HEK293T cells together with a plasmid expressing the miRNA or control sequence. Based on bioinformatics analysis, miR-138 was selected for further analysis and a reporter construct was designed, in which a mutation was produced at the miR-138 putative binding site on the HMMR-AS1 sequence. Consistent with expectations, the HMMR-AS1 mutation abolished miR-138-mediated inhibition of luciferase activity (Fig. 4B-C). Additionally, RNA immunoprecipitation experiments showed that HMMR-AS1 and miR-138 were enriched in Ago2 immunoprecipitation (Fig. 4D). Particularly, miR-138 expression levels were significantly increased with HMMR-AS1 knockdown (Fig. 4E-F). A significant correlation between HMMR-AS1 and miR-138 expression levels in 18 LUAD tissues was revealed through qRT-PCR analysis (Fig. 4G).

### HMMR-AS1 is mediated by the negative regulation of miR-138

**Figure 5:**
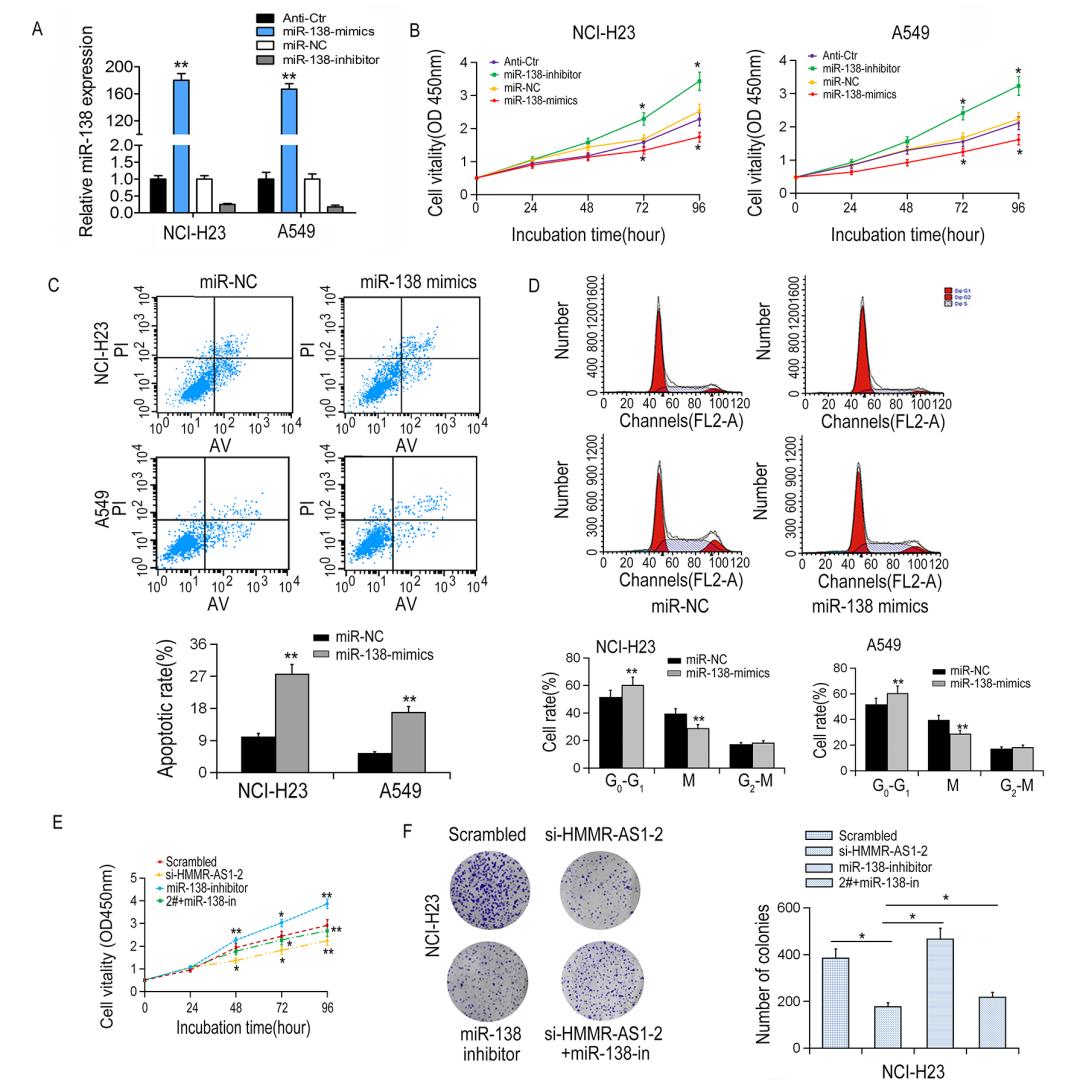
**Effects of miR-138 on proliferation, apoptosis and cell cycle of LUAD cells.** (**A**) MiR-138 expression in NCI-H23 and A549 cells was detected by qRT-PCR after transfection with miR-138 mimics or inhibitor. (**B**) Cell proliferation was inhibited by miR-138 mimics showed by CCK8 assays. (**C, D**) Apoptotic rate and cell cycle arrest in A549 and NCI-H23 cells were detected by flow cytometry. (**E**) NCI-H23 cell viability after co-transfection was determined by CCK8 assay. (**F**) NCI-H23 cell proliferation was determined by colony formation assays. *P < 0.05, **P < 0.01.

We transfected miR-138 mimics/inhibitors into NCI-H23 and A549 cells, respectively, to determine the tumor suppressive role of miR-138 in LUAD cells (Fig. 5A). Results from CCK-8 experiments showed that cell proliferation was significantly reduced by miR-138 overexpression and enhanced by miR-138 inhibition (Fig. 5B). In addition, flow cytometry analysis showed that overexpression of miR-138 induced apoptosis and cell cycle arrest in NCI-H23 and A549 cells (Fig. 5C-D). Particularly, the cell proliferation inhibition mediated by si-HMMR-AS1-2 can be partially reversed by co-transfection with miR-138 inhibitor (Fig. 5E-F), suggesting that HMMR-AS1 promotes cell proliferation via inhibiting miR-138 expression.

### SIRT6 is a target of miR-138 indirectly regulated by HMMR-AS1

**Figure 6:**
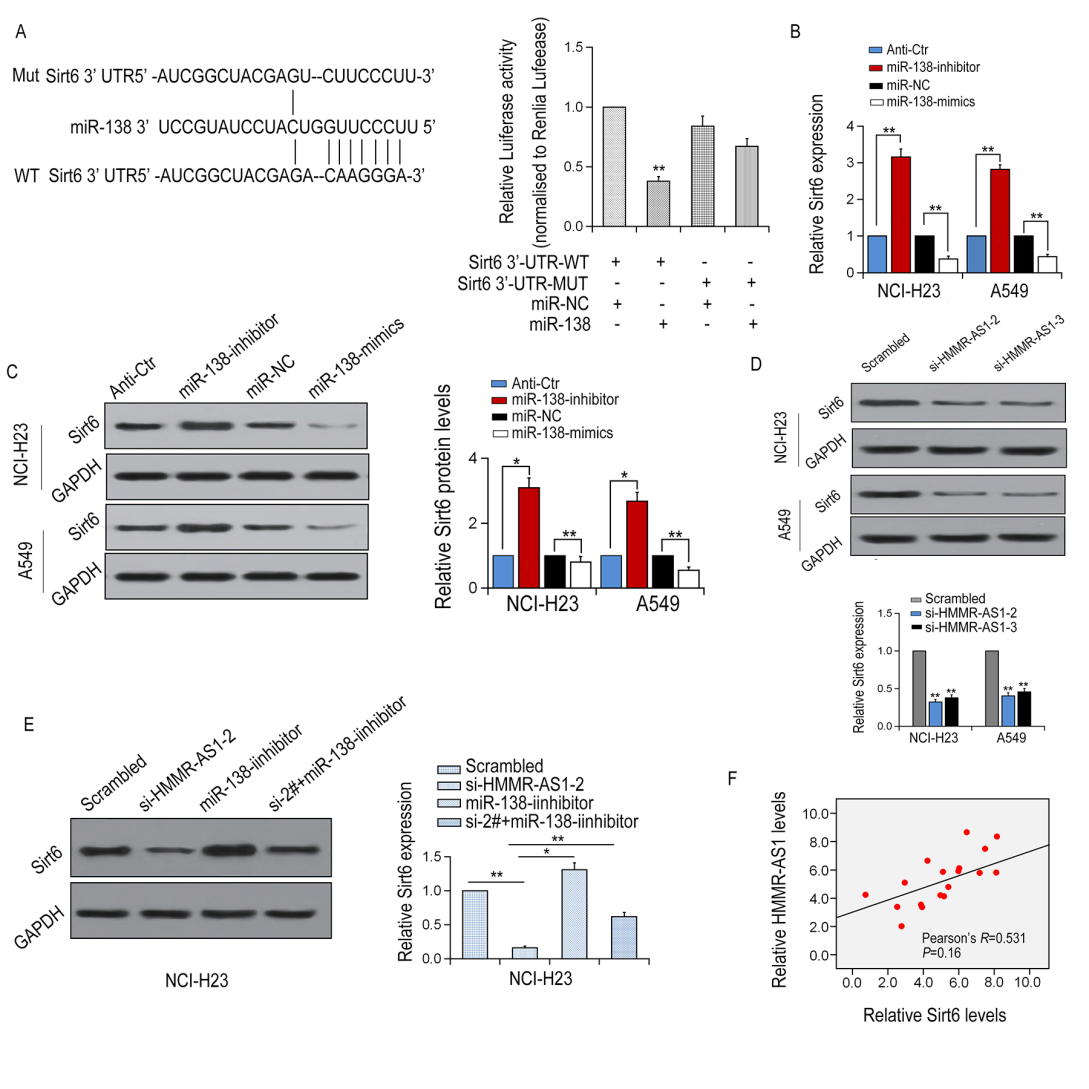
**SIRT6 is a target of miR-138 and is inhibited by HMMR-AS1 knockdown.** (**A, B**) Schematic view of miR-138 putative binding site in the WT/Mut 3′-UTR of SIRT6. Luciferase activity assay in HEK-293T cells transfected with luciferase report plasmids with WT/Mut SIRT6 3′-UTR or miR-138. (**C, D**) Relative mRNA and protein levels of SIRT6 in NCI-H23 and A549 cells after transfection. (**E**) SIRT6 mRNA and protein level in NCI-H23 and A549 cells after HMMR-AS1 knockdown. (**F**) SIRT6 mRNA and protein level in NCI-H23 cells after HMMR-AS1 knockdown and/or miR-138 inhibition. F. Correlation between HMMR-AS1 and SIRT6 expressions in 18 paired LUAD tissues. *P < 0.05, **P < 0.01.

Next, we applied TargetScan and miRanda to predict potential target genes for miR-138. SIRT6 was selected as a potential target gene for miR-138 in LUAD for further study. It was revealed that the 3'UTR region of SIRT6 contains a potential miR-138 binding site. Then, a luciferase reporter gene assay was performed with the wild-type or mutant 3'UTR sequence of the SIRT6 gene. The above plasmid was co-transfected with or without HER-138 mimic into HEK293T cells. It was indicated that compared with the control group, luciferase expression in the SIRT6 3'UTR-WT group was significantly reduced by the co-transfection with miR-138 mimic, whereas the inhibition was neutralized by SIRT6 3'UTR-Mut (Fig. 6A). SIRT6 mRNA and protein expression levels were significantly decreased or increased via overexpression or inhibition of miR-138, respectively, (Figures 6B-D). Besides, SIRT6 mRNA and protein expression levels were significantly reduced with HMMR-AS1 knockdown in NCI-H23 and A549. SIRT6 protein expression inhibition, induced by HMMR-AS1-2 knockdown, was effectively reversed by miR-138 inhibitor (Fig. 6E). We further analyzed the correlation of HMMR-AS1 and SIRT6 expression in 18 pairs of LUAD tissues as well as their matched adjacent non-cancerous tissues, and found a positive correlation between them, which is consistent with the presence of the HMMR-AS1-miR-138-SIRT6 adjustment axis (Figure 6F). Taken together, our data suggest that SIRT6 expression is regulated by HMMR-AS1 via the post-transcriptional regulation of miR-138.

### SIRT6 is up-regulated in LUAD tissues and promotes LUAD cell growth

**Figure 7:**
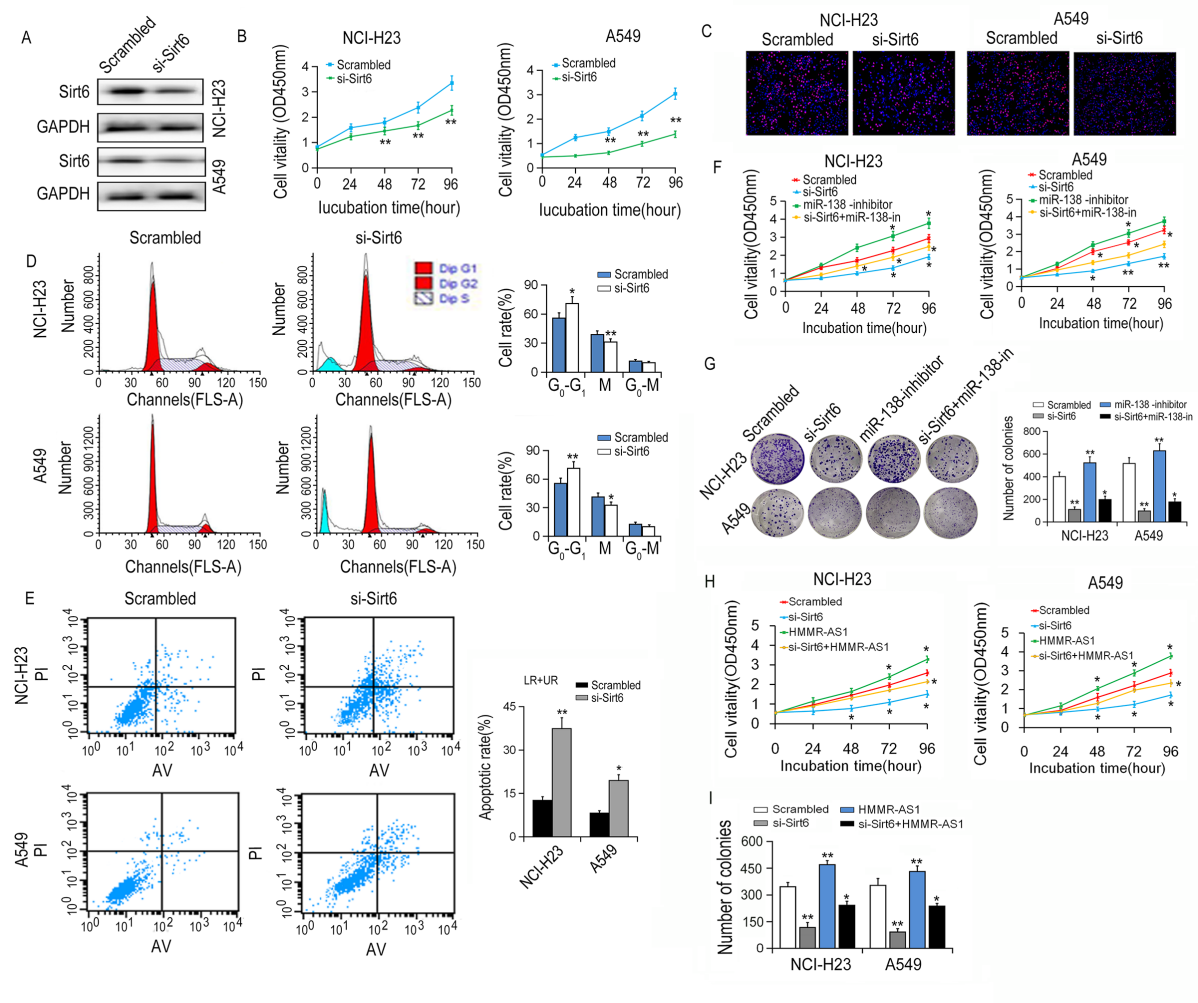
**Effects of SIRT6 knockdown on viability of LUAD cells.** (**A**) The SIRT6 protein expression was determined by western blot after SIRT6 knockdown. (**B, C**) NCI-H23 and A549 cell growth were determined by CCK8 assays and EdU staining assays. (**D**) Cell cycle was analyzed by flow cytometry. (**E**) The apoptotic rates of cells were detected by flow cytometry. (**F, G**) Cell ability of NCI-H23 and A549 cells co-transfection with si-SIRT6 and miR-138 inhibitor was detected by CCK8 and colony formation assays. (**H, I**) Cell ability of NCI-H23 and A549 cells co-transfection with si-SIRT6 and HMMR-AS1 was detected by CCK8 and colony formation assays. *P < 0.05, **P < 0.01.

SIRT6 expression was knocked down after SIRT6 siRNA was transfected into NCI-H23 and A549 cells (Fig. 7A). CCK-8 and EdU incorporation experiments showed that the cell growth activity was significantly reduced after SIRT6 silencing. Similar results were also obtained in colony numbers (Fig. 7B-C). Meanwhile, flow cytometry assay showed that SIRT6 knockdown induced cell cycle arrest and increased apoptosis in NCI-H23 and A549 cells (Fig. 7D-E). Additionally, the proliferation of NCI-H23 and A549 cells was promoted by miR-138 inhibition, and partially reversed through co-transfection with si-SIRT6 (Fig. 7F-G). Similarly, overexpression of HMMR-AS1 increased the proliferation of NCI-H23 and A549 cells, and this effect of HMMR-AS1 was partially abolished by silencing of SIRT6 (Fig. 7H-I).

## DISCUSSION

It was reported recently that lncRNA plays an important role in the carcinogenesis and progression of human cancer [12,13]. In this study, a novel lncRNA HMMR-AS1 associated with LUAD was uncovered, the expression of which in LUAD tissues and cell lines was significantly up-regulated. High expression of HMMR-AS1 was involved in advanced TNM staging, greater tumor volume, as well as positive lymph node metastasis. Additionally, the up-regulation of HMMR-AS1 expression is associated with a shorter OS and PFS in patients with LUAD. HMMR-AS1 knockdown inhibits LUAD cell proliferation and tumor growth, whereas HMMR-AS1 overexpression promotes LUAD cell proliferation. Apoptosis is an important biological process controlling cell growth. We found that knockdown of HMMR-AS1 significantly enhanced the apoptosis in LUAD cells, which maybe the main reason responsible for HMMR-AS1 silencing-mediated inhibition in LUAD cell growth. Additionally, a G0/G1 arrest induced by HMMR-AS1 silencing also partially contribute to the suppressive effect of HMMR-AS1 inhibition on cell proliferation. Together, our results suggest that HMMR-AS1 acts as a carcinogen in the development of LUAD, which can be taken as a potential prognostic factor of LUAD.

Abnormal regulation of these lncRNAs may promote tumor growth and cause uncontrolled tumor growth. For instance, lncRNA 00152 promoted LUAD cell proliferation through interaction with EZH2 and IL24 transcription repression [14]. LncRNA Unigene56159 promotes epithelial mesenchymal transition in hepatocellular carcinoma cells [15]. It was showed in our experimental that the interaction of HMMR-AS1 and miR-138 plays an important role in cancer formation. HMMR-AS1 acts as a molecular sponge of miR-138 in LUAD cells, which plays a role as oncogene. We found that SIRT6 is a potential target of miR-138 via an online prediction database. A luciferase reporter assay was performed to verify that SIRT6 is a direct target of miR-138. It was demonstrated with the assay that miR-138 can be targeted at the 3'UTR region of SIRT6 mRNA. Furthermore, expression of SIRT6 mRNA and protein are inhibited with miR-138 overexpression. It was reported that SIRT6 has characteristics to be both a tumor suppressor and a carcinogenic factor [16]. The expression of SIRT6 is down-regulated in colon cancer, hepatocellular carcinoma, and head and neck squamous cell carcinoma [17]. Down-regulation of SIRT6 expression is associated with higher cancer staging and grades, as well as poor survival rates in cancer patients [18]. In contrast, SIRT6 expression is up-regulated in patients with prostate cancers, which is related to chemoresistance and poor prognosis in patients with cancer [19]. It was found by AZUMA et al. [20] that paclitaxel sensitivity is increased with knockdown of SIRT6 in LUAD cells. In addition, our data initially showed that LUAD cell proliferation is inhibited with SIRT6 knockdown, which induces apoptosis. It was suggested via rescue experiments that SIRT6 knockdown partially reverses the inhibitory effect of miR-138 down-regulation, suggesting that the inhibitory effect of miR-138 on LUAD cell proliferation is dependent on the inhibition of SIRT6 expression.

## Conclusion

In conclusion, we uncovered a novel LUAD-related lncRNA, i.e. HMMR-AS1, and reported for the first time that HMMR-AS1 is a carcinogenic lncRNA that promotes proliferation of human LUAD cells via miR-138-SIRT6 axis, and inhibits apoptosis. This study is of great value for us to understand the function of the lncRNA–miRNA-mRNA ceRNA network in the development of LUAD. HMMR-AS1 could be a potential target for the diagnosis and treatment of LUAD.

## MATERIALS AND METHODS

### Tissue samples and data from TCGA

We obtained 48 paired LUAD and adjacent non-tumor tissues from LUAD patients who were diagnosed based on histopathological evaluation and had underwent surgery at Shanghai Pulmonary Hospital, Tongji University School of Medicine during January 2011 and December 2013. No patients receive chemotherapy or radiotherapy before surgery. All collected tissue samples were immediately frozen in liquid nitrogen and stored at −80°C until required. All subjects provided written informed consent, and all research complied with the principles of the Declaration of Helsinki. The study was approved by the Research Ethics Committee of Shanghai Pulmonary Hospital, Tongji University School of Medicine. A total of 573 entries of LUAD were downloaded from TCGA data portal (https://tcga-data.nci.nih.gov/tcga/). Data exclusion criteria were as follows: (i) duplicate sample data); and (ii) a total survival time of >2,000 days. Overall, 515 LUAD tissues (tumor group) from The Cancer Genome Atlas (TCGA) and 42 adjacent non-cancerous tissues (normal group) were analyzed for the expression of HMMR-AS1.

### Cell culture

Five human LUAD cell lines (A549, NCI-H23, PC-9, C422L, and HCC827) and human normal bronchi epithelium (HBE) cell line were obtained from the American Type Culture Collection (ATCC, Manassas, VA). A549 and HCC827 cells were cultured in RPMI 1640; NCI-H23 and C422L cells were cultured in DMEM; C422L cells were maintained in Dulbecco's modified Eagle medium/F12 (DMEM/F12) (Thermo Fisher Scientific, Waltham, MA, USA) supplemented with 10% fetal bovine serum (FBS) (Corning, NY, USA). NCI-H23 and PC-9 were maintained in RPMI 1640 (Thermo Fisher Scientific) supplemented with 10% FBS. All cells were cultured in a 5% CO_2_ incubator at 37°C.

### qRT-PCR

Total RNA was extracted from frozen tissues or cultured cell lines using TRIZOL reagent (Life Technologies, Carlsbad, CA). The total RNA was reverse-transcribed into cDNA using the ProtoScript® First Strand cDNA Synthesis kit (New England Biolabs, Ipswich, MA, USA). Real-time PCR analyses were performed with SYBR Premix Ex Taq (Takara, Dalian China). Results were normalized to the expression of glyceraldehyde-3-phosphate dehydrogenase (GAPDH). The quantitative reverse transcription-PCR (qRT-PCR) was conducted on the ABI 7500 real-time PCR system (Applied Biosystems, Foster City, CA, USA). The relative expression was standardized by U6 and the multiple changes were measured by 2-ΔΔCt method.

### Cell transfection

LUAD cells were transfected with siRNAs and plasmid vectors using Lipofectamine 2000 (Invitrogen, USA), in accordance with the manufacturer’s protocol. Three HMMR-AS1 siRNAs (si-HMMR-AS1-1, -2, and -3), SIRT6 siRNA, miR-138 mimics, miR-138 inhibitor, and scrambled negative control siRNA (si-NC) were purchased from Invitrogen. Human HMMR-AS1 transcript cDNA (Hankbio, China) and shRNA directed against HMMR-AS1 (Hankbio, China) were inserted into the pCDNA3.1-PLKO vectors, respectively, to construct plasmids pCDNA-HMMR-AS1 and sh-HMMR-AS1. At 48 h post-transfection, cells were harvested for qRT-PCR or western blot analysis.

### Cell proliferation assay

Cell proliferation assay was performed with the CCK-8 kit (Promega). Si-HMMR-AS1 was transfected into NCI-H23 and A549 cells (3000 cells/well), respectively, for 24 hours. Then the transfected cells were seeded into 96-well plates, and cultured in the incubator at 37°C with 5% CO_2_. The relative growth rate of cells was detected with CCK-8 test every 24 hours in accordance with the instruction. In the colony formation assay, 500 NCI-H23 and A549 cells transfected with si-HMMR-AS1, were seeded into 6-well plates and cultured in a suitable medium. Colonies were fixed with methanol 14 days later, and stained with 0.1% crystal violet (Sigma-Aldrich). The number of stained colonies was counted to evaluate the colony formation.

### ISH and evaluation of HMMR-AS1 staining

ISH was performed according to the manufacturer’s protocol (Boster Bio-Engineeting Company, Wuhan, China). Briefly, 4-μm-thick paraffin-embedded sections were deparaffinized with xylene and rehydrated with dilute ethanol of reagent grade. The samples were digested, fixed, hybridized with the 5′-digoxin-labeled probe of HMMR-AS1 at 55°C overnight, and subsequently incubated for 30 min at 4 °C with HRP. Diaminobenzidine was used to develop the stain with a color reaction. The ISH-stained tissue sections were reviewed and scored separately by two blinded pathologists. Scores were determined using a relatively simple, reproducible scoring method based on both the intensity and proportion of HMMR-AS1-positive cells. The staining intensity was scored on a scale of 0–3, as follows: negative (no staining, 0), weak (1), medium (2) or strong (3). The extent of the staining was defined as the percentage of the positive stained areas of tumor cells or normal epithelial cells in relation to the whole tumor area or the entire section of the normal samples, and it was scored on a scale of 0–4 as follows: 0% (0); 1–25% (1); 26–50% (2); 51–75% (3); and 76–100% (4). The sum of the staining-intensity and staining-extent scores was used as the final staining score for HMMR-AS1 (on a scale of 0–7). A final staining score of >3 was considered to denote high-expression of HMMR-AS1.

### Flow cytometry analysis

48 hours after the transfection of si-HMMR-AS1 or si-NC into NCI-H23 and A549 cells, cells were harvested by cell dissociation solution (accutase, Invitrogen, Carlsbad, USA). A flow cytometer (FACScan®; BD Biosciences), equipped with CellQuest software (BD Biosciences), was applied to perform the analysis post Annexin V-FITC/PI double staining. Cells were divided into living cells, dead cells, early apoptotic cells, and late apoptotic cells. The relative ratios of early apoptotic cells to late apoptotic cells of the above transfected cells and control transfectants were compared in each experiment. For cell cycle analysis, cells were subjected to propidium iodide (PI) staining with CycleTEST^TM^ PLUS DNA Kit (BD Biosciences) and analyzed by FACScan flow cytometry in compliance to the protocol. The proportion of cells at G0/G1, S, and G2/M phases was counted and compared.

### EdU assay

Cell proliferation was assessed with the EdU Labeling/Asssay kit (Ribobio, Guangzhou, China) in accordance with the instruction. Briefly, NCI-H23 and A549 cells were seeded into 96-well plates at a density of 5 x 10^3^ cells per well, which were transfected with plasmid DNA or siRNA for 48 hours. Then, 50 μM EdU labeling medium was added to a 96-well plate and cultured for 2 hours in an incubator at 37°C with 5% CO^2^. The cells were stained with anti-EdU working liquid post being treated with 4% paraformaldehyde and 0.5% Triton X-100. The nuclei were stained with DAPI. Following the fluorescence microscopy analysis, five fields were randomly selected from the three wells to calculate the proportion of EdU positive cells.

### Xenograft model *in vivo*

The experiment was approved by the Animal Experimental Ethics Committee of Shanghai Pulmonary Hospital, Tongji University School of Medicine. All experimental procedures with the use of animals are carried out at Shanghai Pulmonary Hospital, Tongji University School of Medicine in strict accordance with the Guidelines for the Management and Use of Laboratory Animals of National Institutes of Health. In the carcinogenicity experiment, control shRNA or sh-HMMR-AS1was consistently transfected to NCI-H23 cells. The lentivirus carrying miR-138, miR-NC (negative control), sh-SIRT6 and blank vector (negative control) were purchased from Genepharma (Shanghai, China). With NCI-H23 cells being infected with lentivirus and screened by puromycin (Sigma, MI, USA), cell lines stably expressing MiR-138 and sh-SIRT6 were established. LUAD cells were injected subcutaneously into the underarm area of either side of male BALB/c nude mice (4-5 weeks old). Tumor size and weight were measured every 3 days and tumor volume was calculated with the formula: V = 0.5 x D x d^2^ (V, volume; D, longest diameter; d, diameter perpendicular to the longest diameter). The mice were euthanized 15 days after the injection, and the growth situation of all tumors subcutaneous were examined. Primary tumors were excised, which was followed by HE staining and immunohistochemical staining according to the methods described in the literature.

### Western blot

NCI-H23 and A549 cells were lysed with RIPA lysis solution (Beyotime) containing a mixture of protease inhibitors. The cell protein lysis product was separated by 10% SDS-PAGE electrophoresis, transferred onto a 0.22 mm PVDF membrane (Millipore), and detected with a specific antibody. A chemiluminescent substrate was added to the specific strip and quantified with densitometry (Quantity One software, BioRad). The GAPDH antibody was used as a control. Anti-Caspase-3 antibody, cleavage Caspase-3, poly (ADP-ribose) polymerase, cleavage PARP, BAX, BAK, cyclin D3 and CDK4 (1:1000) were purchased from Cell Signaling Technology. Anti-SIRT6 antibody was purchased from Abcam. BCL-2 and BCL-XL antibody were purchased from Proteintech.

### RNA immunoprecipitation

RNA immunoprecipitation was performed with the Magna RIP RNA Binding Protein Immunoprecipitation Kit (Millipore, Billerica, MA, USA) according to the instructions. NCI-H23 and A549 cells were lysed with complete RIP lysis buffer and then incubated at 4°C with RIPA buffer ligated to human anti-Argonaute (Ago2) antibody (Millipore). The magnetic beads were washed 6 to 8 hours later, and then incubated with proteinase K at 55°C for 30 minutes. Finally, the immunoprecipitated RNA was applied for qRT-PCR analysis.

### Luciferase assay

DNA fragments containing the wild-type (WT) or mutant (MUT) HMMR-AS1 fragment and the 3' UTR region of SIRT6 were subcloned into the pGL3-Baisc luciferase reporter gene vector (pGL3-Baisc). 50 nM blank vector or miR-148a-5p, miR-138, miR-30d-3p, miR-33b-5p, miR-29c-5p and miR-193a-5p, and 50 ng firefly luciferase reporter gene vectors containing the WT or MUT HMMR-AS1 and 3' UTR region of SIRT6 fragment were co-transfected into human HEK293T cells (1.0 x 104) grown in 24-well plates with Lipofectamie 3000 reagent (Invitrogen, USA). 48 hours after transfection, the luciferase reporter assay was performed with the dual luciferase kit (Promega). Normalization processing was performed to analyze the relative activity of firefly luciferase based on Renilla luciferase. Each transfection trial was repeated for three times.

### Statistical analysis

All statistical analyses were fulfilled via SPSS 17.0 software. Significant differences between groups were analyzed by paired, two-tailed Student's t-test or χ2 test. The effects of variables on survival were determined via univariate and multivariate Cox proportional hazards model. Progression-free survival (PFS) and overall survival (OS) analysis were performed with the Kaplan-Meier method. Correlation between HMMR-AS1, miR-138, and SIRT6 with other clinical factors was calculated with Spearman correlation analysis. P value <0.05 suggested that the difference was of statistical significance.

## SUPPLEMENTARY MATERIAL

Supplementary Table 1

## References

[1] Taylor MD, Bollt O, Iyer SC, Robertson GP. Metastasis suppressor 1 (MTSS1) expression is associated with reduced in-vivo metastasis and enhanced patient survival in lung adenocarcinoma. Clin Exp Metastasis. 2018; 11 10 3041 3054:15–23. 10.1007/s10585-017-9869-3 29218652

[2] Yerukala Sathipati S, Ho SY. Identifying the miRNA signature associated with survival time in patients with lung adenocarcinoma using miRNA expression profiles. Sci Rep. 2017; 7:7507. 10.1038/s41598-017-07739-y 28790336 PMC5548864

[3] Wang X, Li J, Duan Y, Wu H, Xu Q, Zhang Y. Whole genome sequencing analysis of lung adenocarcinoma in Xuanwei, China. Thorac Cancer. 2017; 8:88–96. 10.1111/1759-7714.12411 28083984 PMC5334298

[4] Kopparam J, Chiffelle J, Angelino P, Piersigilli A, Zangger N, Delorenzi M, Meylan E. RIP4 inhibits STAT3 signaling to sustain lung adenocarcinoma differentiation. Cell Death Differ. 2017; 24:1761–71. 10.1038/cdd.2017.81 28574510 PMC5596425

[5] Chandra Gupta S, Nandan Tripathi Y. Potential of long non-coding RNAs in cancer patients: from biomarkers to therapeutic targets. Int J Cancer. 2017; 140:1955–67. 10.1002/ijc.30546 27925173

[6] Forrest ME, Khalil AM. Review: regulation of the cancer epigenome by long non-coding RNAs. Cancer Lett. 2017; 407:106–12. 10.1016/j.canlet.2017.03.040 28400335

[7] Wei MM, Zhou GB. Long Non-coding RNAs and Their Roles in Non-small-cell Lung Cancer. Genomics Proteomics Bioinformatics. 2016; 14:280–88. 10.1016/j.gpb.2016.03.007 27397102 PMC5093404

[8] Chen J, Hu L, Chen J, Pan Q, Ding H, Xu G, Zhu P, Wen X, Huang K, Wang Y. Detection and Analysis of Wnt Pathway Related lncRNAs Expression Profile in Lung Adenocarcinoma. Pathol Oncol Res. 2016; 22:609–15. 10.1007/s12253-016-0046-9 26857641

[9] Li L, Feng T, Qu J, Feng N, Wang Y, Ma RN, Li X, Zheng ZJ, Yu H, Qian B. LncRNA Expression Signature in Prediction of the Prognosis of Lung Adenocarcinoma. Genet Test Mol Biomarkers. 2018; 22:20–28. 10.1089/gtmb.2017.0194 29297704

[10] Liu L, Ni J, He X. Upregulation of the Long Noncoding RNA SNHG3 Promotes Lung Adenocarcinoma Proliferation. Dis Markers. 2018; 2018:5736716. 10.1155/2018/5736716 30154938 PMC6081568

[11] Zhang PP, Wang YQ, Weng WW, Nie W, Wu Y, Deng Y, Wei P, Xu MD, Wang CF. Linc00152 promotes Cancer Cell Proliferation and Invasion and Predicts Poor Prognosis in Lung adenocarcinoma. J Cancer. 2017; 8:2042–50. 10.7150/jca.18852 28819405 PMC5559966

[12] Ning S, Zhang J, Wang P, Zhi H, Wang J, Liu Y, Gao Y, Guo M, Yue M, Wang L, Li X. Lnc2Cancer: a manually curated database of experimentally supported lncRNAs associated with various human cancers. Nucleic Acids Res. 2016; 44:D980–85. 10.1093/nar/gkv1094 26481356 PMC4702799

[13] Zhang H, Wang Y, Lu J, Zhao Y. Long non-coding RNA LINC00222 regulates GSK3β activity and promotes cell apoptosis in lung adenocarcinoma. Biomed Pharmacother. 2018; 106:755–62. 10.1016/j.biopha.2018.06.165 29990868

[14] Chen QN, Chen X, Chen ZY, Nie FQ, Wei CC, Ma HW, Wan L, Yan S, Ren SN, Wang ZX. Long intergenic non-coding RNA 00152 promotes lung adenocarcinoma proliferation via interacting with EZH2 and repressing IL24 expression. Mol Cancer. 2017; 16:17. 10.1186/s12943-017-0581-3 28109288 PMC5251237

[15] Lv J, Fan HX, Zhao XP, Lv P, Fan JY, Zhang Y, Liu M, Tang H. Long non-coding RNA Unigene56159 promotes epithelial-mesenchymal transition by acting as a ceRNA of miR-140-5p in hepatocellular carcinoma cells. Cancer Lett. 2016; 382:166–75. 10.1016/j.canlet.2016.08.029 27597739

[16] Sebastián C, Zwaans BM, Silberman DM, Gymrek M, Goren A, Zhong L, Ram O, Truelove J, Guimaraes AR, Toiber D, Cosentino C, Greenson JK, MacDonald AI, et al. The histone deacetylase SIRT6 is a tumor suppressor that controls cancer metabolism. Cell. 2012; 151:1185–99. 10.1016/j.cell.2012.10.047 23217706 PMC3526953

[17] Geng CH, Zhang CL, Zhang JY, Gao P, He M, Li YL. Overexpression of Sirt6 is a novel biomarker of malignant human colon carcinoma. J Cell Biochem. 2018; 119:3957–67. 10.1002/jcb.26539 29227545

[18] Zhu B, Yan Y, Shao B, Tian L, Zhou W. Downregulation of SIRT6 is associated with poor prognosis in patients with non-small cell lung cancer. J Int Med Res. 2018; 46:1517–27. 10.1177/0300060517750298 29363378 PMC6091845

[19] Liu Y, Xie QR, Wang B, Shao J, Zhang T, Liu T, Huang G, Xia W. Inhibition of SIRT6 in prostate cancer reduces cell viability and increases sensitivity to chemotherapeutics. Protein Cell. 2013; 4:702–10. 10.1007/s13238-013-3054-5 23982738 PMC4875531

[20] Azuma Y, Yokobori T, Mogi A, Altan B, Yajima T, Kosaka T, Onozato R, Yamaki E, Asao T, Nishiyama M, Kuwano H. SIRT6 expression is associated with poor prognosis and chemosensitivity in patients with non-small cell lung cancer. J Surg Oncol. 2015; 112:231–37. 10.1002/jso.23975 26180037

