# Supplementary material for: LncRNA HMMR-AS1 promotes proliferation and metastasis of lung adenocarcinoma by regulating MiR-138/sirt6 axis
Source: Aging (Albany NY). 2019 May 25;11(10):3041–54. doi: 10.18632/aging.101958 (PMC6555459; doi:10.18632/aging.101958)
Supplement: Supplementary Table 1 [file aging-11-101958-s001.pdf]

Table S1. Correlation between HMMR-AS1 and clinicopathological features in 48 LUAD patients.

Parameters	*n*	HMMR-AS1		P
		High	Low	
Age (years)				0.585
>60	30	18	12	
≤60	18	12	6	
Gender				0.752
Male	29	19	10	
Female	19	11	8	
Tumor diameter (cm)				0.038*
≥5cm	17	14	3	
<5cm	31	22	9	
TNM stage				0.684*
I	2	2	0	
II	14	8	7	
III	18	16	2	
IV	16	10	6	
Lymphatic metastasis				0.007*
Y	23	16	7	
N	25	19	6	
Invasion				0.026*
Y	6	5	1	
N	42	31	11	
Tumor differentiation				0.152
High	8	18	7	
Middle	38	22	16	
Low	2	2	0	

* Chi-squared test. *$P<0.05$
